# A Semiparametric Bayesian Approach to Heterogeneous Spatial Autoregressive Models

**DOI:** 10.3390/e26060498

**Published:** 2024-06-07

**Authors:** Ting Liu, Dengke Xu, Shiqi Ke

**Affiliations:** School of Economics, Hangzhou Dianzi University, Hangzhou 310018, China; 22150107@hdu.edu.cn (T.L.); 231150084@hdu.edu.cn (S.K.)

**Keywords:** heterogeneous semiparametric spatial autoregressive models, Bayesian estimate, Gibbs sampler, Metropolis–Hastings algorithm, B-spline

## Abstract

Many semiparametric spatial autoregressive (SSAR) models have been used to analyze spatial data in a variety of applications; however, it is a common phenomenon that heteroscedasticity often occurs in spatial data analysis. Therefore, when considering SSAR models in this paper, it is allowed that the variance parameters of the models can depend on the explanatory variable, and these are called heterogeneous semiparametric spatial autoregressive models. In order to estimate the model parameters, a Bayesian estimation method is proposed for heterogeneous SSAR models based on B-spline approximations of the nonparametric function. Then, we develop an efficient Markov chain Monte Carlo sampling algorithm on the basis of the Gibbs sampler and Metropolis–Hastings algorithm that can be used to generate posterior samples from posterior distributions and perform posterior inference. Finally, some simulation studies and real data analysis of Boston housing data have demonstrated the excellent performance of the proposed Bayesian method.

## 1. Introduction

In recent decades, spatial data analysis based on spatial autoregressive (SAR) models has become a very important and popular research direction in the academic research of econometricians and statisticians. Among them, in-depth research has been conducted on inference theories and methods based on linear SAR models and their extensions, including estimation, variable selection, hypothesis testing, etc., and a large amount of literature has also been produced, such as [1,2,3]. Specifically, there are many in-depth research achievements on linear SAR models, such as [4,5,6,7]. However, in spatial data analysis, there is often a nonlinear relationship between response variables and covariates. Therefore, in order to explore this complex phenomenon, some semiparametric SAR models have been proposed in recent years and have been thoroughly studied. For example, based on partially linear spatial autoregressive models, Su and Jin [8] proposed a profile quasi-maximum likelihood estimation method and established asymptotic theoretical properties of the obtained estimators. By using the spline approximations and instrumental variables estimation method, Du et al. [9] developed an estimation method for partially linear additive spatial autoregressive models, and derived asymptotic theoretical properties of the obtained estimators. For partially linear single-index spatial autoregressive models, Cheng and Chen [10] developed an estimation method and established consistency and asymptotic normality of the estimators under some mild assumptions. Other related research results on semiparametric SAR models can also be found in [11,12]. Previous research on various spatial autoregressive models was mainly based on the assumption of homoscedasticity, which assumes that the variance of model errors is constant. As is well known, heteroscedasticity is a common phenomenon in spatial data analysis. Therefore, using statistical inference methods under the assumption of homogeneity may lead to erroneous inference, as seen in Lin and Lee [13]. Therefore, it is necessary to study heterogeneous spatial autoregressive models. Especially in recent years, many researchers have conducted in-depth research on spatial autoregressive models where the error variance is heteroscedasticity. For example, SAR models with heteroscedasticity was studied by Dai et al. [14] for Bayesian local influence analysis. However, existing literature on heterogeneous spatial autoregressive models assume that the variance term is fixed and does not perform regression modeling analysis like the mean. In addition, in many application fields, such as econometrics, the assumption of equal variance may not be suitable for modeling data that exhibit heteroscedasticity. Therefore, we propose a new model, called a heterogeneous semiparametric spatial autoregressive model, in which the variance parameters are allowed to be modeled by using some covariates.

In addition, many estimation methods have recently been developed for SAR models from both frequentist and Bayesian perspectives. Specifically, due to the rapid development of advanced computing technology in the era of big data, Bayesian statistical analysis of SAR models and various other statistical models has received increasing attention, and large quantities of related research achievements have emerged in recent years. For example, within the framework of longitudinal data and for the generalized partial linear mixed models, Tang and Duan [15] studied an effective semiparametric Bayesian method. By using spline approximation, Xu and Zhang [16] introduced a Bayesian method for the partially linear model with heteroscedasticity based on the variance modelling technique. Based on the assumption that the response variables and random effects follow multivariate skew-normal distributions, a new spatial dynamic panel data model was proposed by Ju et al. [17] and a Bayesian local influence analysis method was developed to simultaneously evaluate the impact of small perturbations on the data, priors, and sampling distributions. Pfarrhofer and Piribauer [18] studied Bayesian variable selection for high-dimensional spatial autoregressive models based on two shrinkage priors. Wang and Tang [19] made Bayesian statistical inference based on a quantile regression model with nonignorable missing covariates. To capture the linear and nonlinear relationships between explanatory variables and their responses to spatially relevant data, Chen and Chen [20] developed a Bayesian sampling-based method based on the partially linear single-index spatial autoregressive models, in which it includes an efficient MCMC approach and explores the joint posterior distributions by using a Gibbs sampler. Within the framework of longitudinal data, Zhang et al. [21] proposed semiparametric mixed-effects double regression models for analysis based on spline approximation technology, in which they jointly modeled the mean and variance of the mixed-effects as a function of covariates. To our knowledge, there is not much work on semiparametric Bayesian methods for heterogeneous spatial autoregressive models due to their complex spatial correlation structures. Therefore, based on a hybrid effective algorithm that combines a Gibbs sampler and the Metropolis–Hastings algorithm and has the advantages of both algorithms, this paper develops a Bayesian method for heterogeneous semiparametric spatial autoregressive models based on variance modeling.

The outline of the paper is as follows. A new heterogeneous SSAR model is introduced in Section 2. In Section 3, we derive the full conditional distributions for implementing the sampling-based method, and develop a Bayesian method to obtain estimates by using a Gibbs sampler and the Metropolis–Hastings algorithm. Section 4 presents some simulation studies to illustrate the proposed methodology. As an application example, Section 5 analyzes the Boston house price data by using the proposed method. A brief conclusion and discussion is given in Section 6.

## 2. Heterogeneous Semiparametric Spatial Autoregressive Models

As is well known, the form of classical semiparametric spatial autoregressive models is as follows:

(1) Y=ρWY+Xβ+g(U)+ε,

where $Y={\left(y_{1},y_{2},\cdots ,y_{n}\right)}^{T}$ is an *n*-dimensional response variable, |ρ|<1 is an unknown spatial lag parameter that reflects spatial autocorrelation between neighbors, and *W* is a known spatial weight matrix with zero diagonal elements. In the mean model, $X={\left(x_{1},x_{2},\cdots ,x_{n}\right)}^{T}$ is an n×p explanatory variable matrix where the *i*th row is $x_{i}^{T}=\left(x_{i1},\cdots ,x_{ip}\right)$ and $\beta ={\left(\beta_{1},\cdots ,\beta_{p}\right)}^{T}$ is a *p*-dimensional unknown regression coefficient to be estimated; moreover, g(·) is an arbitrary unknown smooth function in the mean model, which needs to be estimated; $U={\left(u_{1},u_{2},\cdots ,u_{n}\right)}^{T}$ is an *n*-dimensional vector whose *i*th row $u_{i}$ is an univariate observed covariate; ε is an *n*-dimensional vector that represents the regression errors of an independent and identically distributed regression disturbances with zero mean and finite variance $\sigma^{2}$.

In addition, according to Xu and Zhang [16], this paper considers the heterogeneity of the variance in the model and assumes that the variance parameters are related to other explanatory variables; thus, we establish a regression model for the variance parameters, namely

(2) $$\sigma_{i}^{2}=h\left(z_{i}^{T}\gamma \right),\;$$

where $z_{i}^{T}=\left(z_{i1},\cdots ,z_{iq}\right)$ is an explanatory variable vector related to the variance of $\varepsilon_{i}$ and $\gamma ={\left(\gamma_{1},\cdots ,\gamma_{q}\right)}^{T}$ is a *q*-dimensional unknown regression coefficient to be estimated in the variance model. Some elements in $z_{i}$ may coincide with some elements in $x_{i}$. In addition, for the identifiability of the models and considering that the variance is positive, h(·) is a known monotonic positive function. For example, exponential functions are often used to model the variance. So, heterogeneous SSAR models are considered in this paper as follows:

(3) $$\left\{\begin{array}{c} Y=\rho WY+X\beta +g(U)+\varepsilon ,\\ \varepsilon ∼N(0,\Sigma ),\\ \Sigma =diag(\sigma_{1}^{2},\sigma_{2}^{2},\cdots ,\sigma_{n}^{2})\\ \sigma_{i}^{2}=\exp\left(z_{i}^{T}\gamma \right),\\ i=1,2,\cdots ,n. \end{array}\right.\;$$

## 3. Bayesian Inference

### 3.1. B-Splines for the Nonparametric Function

From model (3), we obtain the log-likelihood function

(4) $$\ell_{n}\left(\rho ,\beta ,\gamma |Y,X,Z,U\right)=-\frac{n}{2}ln\left(2\pi \right)-\frac{1}{2}\sum_{i=1}^{n}z_{i}^{T}\gamma +ln\left|A\right|-\frac{1}{2}e^{T}\Sigma^{-1}e,\;$$

where $e=AY-X\beta -g\left(U\right),A=I_{n}-\rho W$, and $I_{n}$ is an (n×n) identity matrix.

There are now a large number of nonparametric techniques for handling nonparametric functions g(·) in (3), such as the smoothing splines and the kernel methods. This paper considers B-spline approximation to transform g(·) into a linear function composed of a set of basis functions. It can be summarized as follows: $\{s_{i}\},i=1,\cdots ,n$ performs a partition on the interval [0, 1], which is called as the internal knots and satisfies $0=s_{0}<s_{1}<\cdots <s_{k_{n}}<s_{k_{n}+1}=1.$ This results in $K=K_{n}+l$ normalized B-spline basis functions of order *l*, which form the basis of a linear spline space. The main reason for using B-splines here is because they have advantages such as bounded support and numerical stability. As well as we know, selection of knots is usually an important aspect that cannot be ignored in the implementation process of B-splines. In this paper, our main focus is inference on the parameters in the mean model and the variance model. Therefore, by following the idea of Zhang et al. [21], the number of internal knots is selected as the integer part of $n^{1/5}$. Thus, $\pi^{T}\left(u\right)\alpha$ is used to approximate g(u), in which $\pi \left(u\right)={\left(B_{1}\left(u\right),\ldots ,B_{K}\left(u\right)\right)}^{T}$ is a basis function vector and $\alpha \in R^{K}$. In this way, we can linearize the nonparametric function g(·) in (3) as follows:

(5) $$g\left(u_{i}\right)\approx \pi^{T}\left(u_{i}\right)\alpha .\;$$

Thus, based on (5), we can rewritten the likelihood function (4) as follows:

(6) $$\ell_{n}\left(\rho ,\beta ,\gamma |Y,X,Z,U\right)=-\frac{n}{2}ln\left(2\pi \right)-\frac{1}{2}\sum_{i=1}^{n}z_{i}^{T}\gamma +ln\left|A\right|-\frac{1}{2}{\left(AY-X\beta -B\alpha \right)}^{T}\Sigma^{-1}\left(AY-X\beta -B\alpha \right),\;$$

where $B={\left(\pi \left(u_{1}\right),\pi \left(u_{2}\right),\cdots ,\pi \left(u_{n}\right)\right)}^{T}.$

### 3.2. Prior Selection of Parameters

This paper will use a Bayesian approach to estimate unknown parameters ρ, β,α and γ. Thus, to obtain Bayesian estimation, the prior distributions of unknown parameters to be estimated in the model should be given first. For the convenience of algorithm implementation, normal prior distributions are often chosen as $\beta ∼N(\beta_{0},b_{\beta })$, $\alpha ∼N(\alpha_{0},\tau^{2}I_{K})$,$\gamma ∼N(\gamma_{0},B_{\gamma })$, and ρ∼U(−1,1), in which $\beta_{0},\alpha_{0},\gamma_{0}$ and $b_{\beta },B_{\gamma }$ are known hyperparameter vectors or matrices. Moreover, the prior distribution of $\tau^{2}$ is the $IG(a_{\tau },b_{\tau })$, and its density function is

$$p\left(\tau^{2}|a_{\tau },b_{\tau }\right)\propto {\left(\tau^{2}\right)}^{-a_{\tau }-1}\exp\left(-b_{\tau }/\tau^{2}\right),$$

in which $a_{\tau }$ and $b_{\tau }$ are assumed to be positive constants and known. In this paper, we mainly focus on the case where the prior distribution of model parameters is a normal distribution. However, the proposed computational algorithm is also applicable to other specific prior distributions.

### 3.3. Posterior Inference

Let θ=(β,α,γ,ρ) and then we aim to estimate the unknown parameters of θ. Based on the proposed model (3) and Gibbs sampling, the specific sampling process is carried out according to the following steps by sampling from joint posterior distribution p(θ|Y,X,Z,U).

**Step 1.** The initial values of parameters are set as $\theta^{\left(0\right)}=\left(\beta^{\left(0\right)},\alpha^{\left(0\right)},\gamma^{\left(0\right)},\rho^{\left(0\right)}\right).$

**Step 2.** Compute $\Sigma^{\left(l\right)}=\mathrm{diag}\left\{\exp\left(z_{i}^{T}\gamma^{\left(l\right)}\right)\right\}$ and $A^{\left(l\right)}=I_{n}-\rho^{\left(l\right)}W$ on the basis of $\theta^{\left(l\right)}=\left(\beta^{\left(l\right)},\alpha^{\left(l\right)},\gamma^{\left(l\right)},\rho^{\left(l\right)}\right)$.

**Step 3.** Based on $\theta^{\left(l\right)}=\left(\beta^{\left(l\right)},\alpha^{\left(l\right)},\gamma^{\left(l\right)},\rho^{\left(l\right)}\right),$ sample $\theta^{\left(l+1\right)}=\left(\beta^{\left(l+l\right)},\alpha^{\left(l+1\right)},\gamma^{\left(l+l\right)},\rho^{\left(l+l\right)}\right)$ as follows:

- Sampling $\tau^{2(l+1)}$ from the conditional distribution below:

(7) $$p\left(\tau^{2}|\alpha^{\left(l\right)}\right)\propto {\left(\tau^{2}\right)}^{-\frac{K}{2}-a_{\tau }-1}\exp\left\{-\frac{{\left(\alpha^{\left(l\right)}-\alpha_{0}\right)}^{T}\left(\alpha^{\left(l\right)}-\alpha_{0}\right)+2b_{\tau }}{2\tau^{2}}\right\}.\;$$

- Sampling $\alpha^{\left(l+1\right)}$ from the conditional distribution below:
  (8) $$p\left(\alpha |Y,X,Z,U,\gamma^{\left(l\right)},\rho^{\left(l\right)}\right)∼N\left({\tilde{\mu }}_{\alpha },{\tilde{\Sigma }}_{\alpha }\right),\;$$
  where ${\tilde{\mu }}_{\alpha }={\tilde{\Sigma }}_{\alpha }\left(\tau^{-2(l+1)}I_{K}\alpha_{0}+B^{T}{\Sigma^{\left(l\right)}}^{-1}\left(A^{\left(l\right)}Y-X\beta^{\left(l\right)}\right)\right)$ and ${\tilde{\Sigma }}_{\alpha }={\left(\tau^{-2(l+1)}I_{K}+B^{T}{\Sigma^{\left(l\right)}}^{-1}B\right)}^{-1}.$

- Sampling $\beta^{\left(l+1\right)}$ from the conditional distribution below:
  (9) $$p\left(\beta |Y,X,Z,U,\alpha^{\left(l+1\right)},\gamma^{\left(l\right)},\rho^{\left(l\right)}\right)∼N\left({\tilde{\mu }}_{\beta },{\tilde{\Sigma }}_{\beta }\right),\;$$
  where ${\tilde{\mu }}_{\beta }={\tilde{\Sigma }}_{\beta }\left(b_{\beta }^{-1}\beta_{0}+X^{T}{\Sigma^{\left(l\right)}}^{-1}\left(A^{\left(l\right)}Y-B\alpha^{\left(l+1\right)}\right)\right)$ and ${\tilde{\Sigma }}_{\beta }={\left(b_{\beta }^{-1}+X^{T}{\Sigma^{\left(l\right)}}^{-1}X\right)}^{-1}.$

- Sampling $\gamma^{\left(l+1\right)}$ from the conditional distribution below:
  (10) $$\begin{array}{cc} p(\gamma |Y,X,Z,U,\beta^{\left(l+1\right)},\alpha^{\left(l+1\right)},\rho^{\left(l\right)})\propto & {\left|\Sigma \right|}^{-\frac{1}{2}}\exp\{-\frac{1}{2}e^{(l,l+1),T}\Sigma^{-1}e^{\left(l,l+1\right)}\\ \;\;\;\;\;-\frac{1}{2}{\left(\gamma -\gamma_{0}\right)}^{T}B_{\gamma }^{-1}\left(\gamma -\gamma_{0}\right)\}. & \end{array}$$
  where $e^{\left(l,l+1\right)}=A^{\left(l\right)}Y-X\beta^{\left(l+1\right)}-B\alpha^{\left(l+1\right)}.$

- Sampling $\rho^{\left(l+1\right)}$ from the conditional distribution below:
  (11) $$p\left(\rho |Y,X,Z,U,\beta^{\left(l+1\right)},\alpha^{\left(l+1\right)},\gamma^{\left(l+1\right)}\right)\propto \left|A\right|\exp\left\{-\frac{1}{2}e^{(l+1)T}{\Sigma^{\left(l+1\right)}}^{-1}e^{\left(l+1\right)}\right\}.\;$$
  where $e^{\left(l+1\right)}=A^{\left(l+1\right)}Y-X\beta^{\left(l+1\right)}-B\alpha^{\left(l+1\right)}.$

**Step 4.** Repeating Steps 2 and 3.

According to the steps of the above algorithm, we can easily obtain the sample sequences $(\beta^{\left(t\right)},\alpha^{\left(t\right)},\gamma^{\left(t\right)},\rho^{\left(t\right)}),t=1,2,\cdots$. It is easy to find that the fully conditional distributions $p\left(\tau^{2}|\alpha^{\left(l\right)}\right),p\left(\beta |Y,X,Z,U,\alpha^{\left(l\right)},\gamma^{\left(l\right)},\rho^{\left(l\right)}\right)$ and $p(\alpha |Y,X,Z,U,\beta^{\left(l+1\right)},\gamma^{\left(l\right)},\rho^{\left(l\right)})$ are Inverse Gamma and normal distributions, respectively, and extracting observations from these familiar standard distributions is fast and easy. Unfortunately, fully conditional distributions $p(\gamma |Y,X,Z,U,\beta^{\left(l+1\right)},\alpha^{\left(l+1\right)},\rho^{\left(l\right)})$ and $p(\rho |Y,X,Z,U,\beta^{\left(l+1\right)},\alpha^{\left(l+1\right)},\gamma^{\left(l+1\right)})$ are nonstandard distributions and appear quite complex, making it quite difficult to extract random numbers from these conditional distributions. To solve the problem of difficult sampling of these posterior distributions, the Metropolis–Hastings algorithm is used. In order to sample from (10) by Metropolis–Hastings algorithm, the normal distribution $N(0,\sigma_{\gamma }^{2}\Omega_{\gamma }^{-1})$ is chosen as the proposal distribution, in which we should choose a suitable $\sigma_{\gamma }^{2}$ so that the average acceptance rate is approximately between 0.25 and 0.45 (Gelman et al. [22]), and take

$$\Omega_{\gamma }=\frac{1}{2}\sum_{i=1}^{n}\frac{e_{i}^{2}}{\exp\{z_{i}^{T}\gamma \}}z_{i}z_{i}^{T}+B_{\gamma }^{-1}.$$

The process of implementing the Metropolis–Hastings algorithm is as follows: assuming that the current value is $\gamma^{\left(l\right)}$ at the (l+1)th iteration, we should generate a new candidate value $\gamma^{*}$ from $N(\gamma^{\left(l\right)},\sigma_{\gamma }^{2}\Omega_{\gamma }^{-1})$ and then decide whether to accept it based on the following probability:

$$\min\left\{1,\frac{p(\gamma^{*}|Y,X,Z,U,\beta^{\left(l+1\right)},\alpha^{\left(l+1\right)},\rho^{\left(l\right)})}{p(\gamma^{\left(l\right)}|Y,X,Z,U,\beta^{\left(l+1\right)},\alpha^{\left(l+1\right)},\rho^{\left(l\right)})}\right\}.$$

In addition, according to [18], the Bayesian estimate of ρ is obtained based on a Metropolis-within-Gibbs step and by using sampling observations from (11).

Based on the observation results generated by the above calculation algorithm, the Bayesian estimation of the unknown parameter (β,α,γ,ρ) can be obtained. Specifically, it is assumed that the observation value $\left\{\left(\beta^{\left(j\right)},\alpha^{\left(j\right)},\gamma^{\left(j\right)},\rho^{\left(j\right)}\right):j=1,2,\cdots ,J\right\}$ of (β,α,γ,ρ) generated from the joint conditional distribution p(β,α,γ,ρ|Y,X,Z,U) is obtained by using the hybrid algorithm proposed earlier. Then define Bayesian estimators of the unknown parameters (β,α,γ,ρ) as follows:

$$\hat{\beta }=\frac{1}{J}\sum_{j=1}^{J}\beta^{\left(j\right)},\hat{\alpha }=\frac{1}{J}\sum_{j=1}^{J}\alpha^{\left(j\right)},\hat{\gamma }=\frac{1}{J}\sum_{j=1}^{J}\gamma^{\left(j\right)},\hat{\rho }=\frac{1}{J}\sum_{j=1}^{J}\rho^{\left(j\right)}.$$

Similar to Geyer [23], it is not difficult to prove that $\left(\hat{\beta },\hat{\alpha },\hat{\gamma },\hat{\rho }\right)$ are consistent estimates of their corresponding posterior means. In addition, we use the “leave-one-out” technique to obtain Bayesian estimates of posterior covariance matrices. For example, the specific formula of the estimator for $\mathrm{Var}(\beta |\theta_{\beta^{-}},Y,X,Z,U)$ can be expressed as follows:

$$\hat{\mathrm{Var}}\left(\beta |\theta_{\beta^{-}},Y,X,Z,U\right)={\left(J-1\right)}^{-1}\sum_{j=1}^{J}\left(\beta^{\left(j\right)}-\hat{\beta }\right){\left(\beta^{\left(j\right)}-\hat{\beta }\right)}^{T},$$

in which $\theta_{\beta^{-}}$ denotes the parameter θ excluding β. A similar approach can be used to obtain the estimators of $\mathrm{Var}(\alpha |\theta_{\alpha^{-}},Y,X,Z,U)$, $\mathrm{Var}(\rho |\theta_{\rho^{-}},Y,X,Z,U)$, and $\mathrm{Var}(\gamma |\theta_{\gamma^{-}},Y,X,Z,U)$.

## 4. Simulation Study

This section conducts a simulation study to evaluate the performance of the proposed Bayesian method under different sample sizes, spatial parameter values, and prior information selections. According to Lee [24] and Xie et al. [7], let n=R×m and $W=I_{R}⊗H_{m}$ be the weight matrix, in which ⊗ represents the Kronecker product and $H_{m}=\left(l_{m}l_{m}^{T}-I_{m}\right)/\left(m-1\right)$, $l_{m}$ is an *m*-dimensional vector with all component elements being 1. In order to investigate whether the proposed Bayesian estimation method is sensitive to the selection of prior distributions, we consider three hyperparameter values in the prior distributions of unknown parameters β,α,γ,ρ:

Type I: $\beta_{0}={\left(1,-0.5,0.5\right)}^{T},b_{\beta }=0.25\times I_{3}$, $\gamma_{0}={\left(1,-0.5,0.5\right)}^{T},B_{\gamma }=0.25\times I_{3}$, $\alpha_{0}={\left(0,\cdots ,0\right)}^{T}$, $a_{\tau }=1,b_{\tau }=1.$ This situation can be considered as having good prior information.

Type II: $\beta_{0}={\left(0,0,0\right)}^{T},b_{\beta }=I_{3}$,$\gamma_{0}={\left(0,0,0\right)}^{T},B_{\gamma }=I_{3}$, $\alpha_{0}={\left(0,\cdots ,0\right)}^{T}$, $a_{\tau }=1,b_{\tau }=1.$ This hyperparameter situation is considered to have no prior information.

Type III: $\beta_{0}=3\times {\left(1,-0.5,0.5\right)}^{T},b_{\beta }=10\times I_{3}$,$\gamma_{0}=3\times {\left(1,-0.5,0.5\right)}^{T},B_{\gamma }=10\times I_{3}$, $\alpha_{0}={\left(0,\cdots ,0\right)}^{T}$, $a_{\tau }=1,b_{\tau }=1.$ This can be seen as a situation where the previous information was inaccurate.

In this section, *R* is selected as 25, 50, 75 and *m* is set to 4, and thus, *n* is to be 100, 200, 300. Furthermore, we generate *X* and *Z*, respectively, from the multivariate normal distribution with zero mean vector and covariance matrix $\Sigma_{0}=\left(c_{ij}\right)$ where $c_{ij}=0.5^{\left|i-j\right|},i=1,\cdots ,p\left(q\right),j=1,\cdots ,p\left(q\right)$. Moreover, to reflect the different spatial dependencies between response variables, spatial parameters ρ=−0.5,0,0.5 are selected to represent different spatial dependencies; $\beta ={\left(1,-0.5,0.5\right)}^{T}$, $g\left(u_{i}\right)=0.5\sin\left(2\pi u_{i}\right)$ where $u_{i}$ follows a uniform distribution U(0,1),and the structure of the variance model is $\log\left(\sigma_{i}^{2}\right)=z_{i}^{T}\gamma$ with $\gamma ={\left(1,-0.5,0.5\right)}^{T}$.

Based on the various parameter setting environments and generated datasets mentioned above, we use the hybrid MCMC algorithm based on 100 replications to evaluate Bayesian estimations of unknown parameters under different sample sizes. Checking whether the MCMC sampler converges in algorithm implementation is an important thing. Therefore, here the estimated potential scale reduction (EPSR) value is used to diagnose whether the MCMC algorithm converges for each dataset [25]. It can be easily observed that in all the runs we are considering, the EPSR value is very close to 1 and less than 1.1 after 3000 iterations. Therefore, after discarding the first 3000 burn-in iterations, collect the observation results of the following J=2000 for statistical inference. In addition, to evaluate the performance of the nonparametric function estimation, the square root of average square errors (RASE) are used here as the criterion for evaluation,

$$\mathrm{RASE}\left(\hat{g}\left(u\right)\right)=E{\left\{\frac{1}{n}\sum_{i=1}^{n}{\left[\hat{g}\left(u_{i}\right)-g\left(u_{i}\right)\right]}^{2}\right\}}^{\frac{1}{2}}.$$

**Figure 1:**
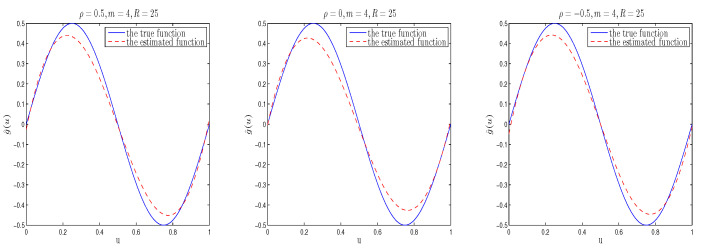
When m=4, R=25,t pye=I, the curve plot of the estimated function and its true values of g(u) based on different ρ’s (the corresponding spatial parameters from left to right are ρ=0.5,0,−0.5).

**Figure 2:**
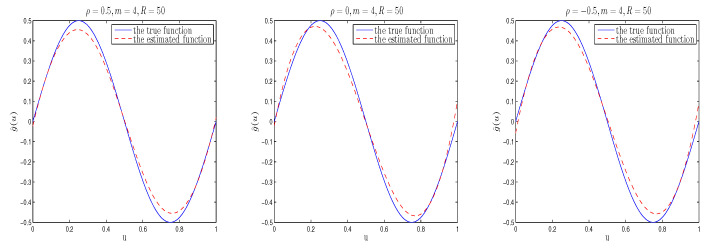
When m=4, R=50, tpye=I, the curve plot of the estimated function and its true values of g(u) based on different ρ’s (the corresponding spatial parameters from left to right are ρ=0.5,0,−0.5).

**Figure 3:**
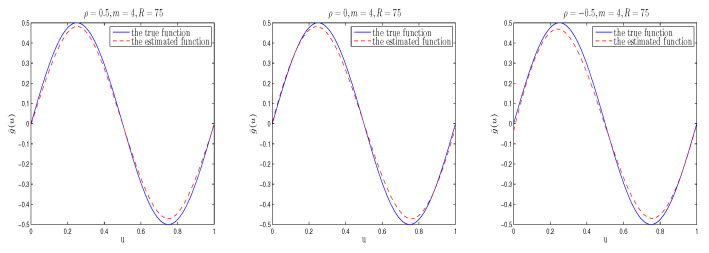
When m=4, R=75, tpye=I, the curve plot of the estimated function and its true values of g(u) based on different ρ’s (the corresponding spatial parameters from left to right are ρ=0.5,0,−0.5).

The simulation results are listed in Table 1, Table 2, Table 3 and Table 4. Moreover, in order to directly examine the accuracy of the estimation of function g(u), we plot the true value of function g(u) and its estimated curve under different cases. To save space, we only list some nonparametric estimation curve results with different spatial parameters in Figure 1, Figure 2 and Figure 3. Figure 1, Figure 2 and Figure 3 depict the real sine curve and its estimated curve based on B-spline approximation. It is easy to observe that all estimated curves are close to the true curve, which indicates that the estimation of g(·) using B-splines in the mean model performs well.

In Table 1, Table 2 and Table 3, “Bias” represents the absolute difference between the true and mean values estimated by Bayesian estimation of parameters based on 100 replicates, and “SD” denotes standard deviation of the Bayesian estimates, while “RMS” is the root mean square between the estimated and true values based on 100 replicates. From Table 1, Table 2 and Table 3, we can see that (i) From the Bias, RMS and SD values of Bayesian estimation, it can be seen that regardless of the prior information input, Bayesian estimation is quite accurate; and for different prior distributions, the proposed estimation method performs well, indicating that Bayesian estimation is not sensitive to prior information input. (ii) Bayesian estimation improves as the sample size increases; (iii)The Bayesian estimation results obtained based on different spatial parameters are similar. (iv) In the same situation, the RMS and SD values of the mean parameters are smaller than that of the variance parameters, which is consistent with the fact that lower order moments are easier to estimate than higher order moments. Furthermore, from Figure 1, Figure 2 and Figure 3, it can be seen that under the considered parameter settings, the estimated function curve is very close to its corresponding true curve, which is consistent with the phenomenon found in Table 4. In summary, the above simulation research results indicate that applying the Bayesian estimation method proposed in this paper to heterogeneous SSAR models is effective.

## 5. Real Data Analysis

Boston housing price data are a commonly used example, and many authors have conducted in-depth analysis based on different statistical models, such as [26,27], and so on. This section will also use the Bayesian estimation method proposed in this paper to analyze these data. This dataset can be easily obtained from R’s spdep library, which includes 14 variables and 506 observations. A detailed explanation of the variables involved in the dataset is presented in Table 5.

In addition, following the variable selection results of Xie et al. [7], we take MEDV as the response variable of the model (represented by *Y*) and the important explanatory variables selected by Xie et al. [7] as the X- variables: CRIM (denoted by $X_{1}$), ZN (denoted by $X_{2}$),NOX (denoted by $X_{3}$), RM(denoted by $X_{4}$), DIS (denoted by $X_{5}$), RAD (denoted by $X_{6}$),TAX (denoted by $X_{7}$), PTRATIO(denoted by $X_{8}$), and B (denoted by $X_{9}$). In order to facilitate data modeling and analysis, all variables were centralized and the index variable was set to $u=\sqrt{LSTAT}$.

In addition, the Euclidean distances calculated using longitude and latitude as [27,28] are used to generate the space weight matrix $W=(w_{ij})$, where

$$w_{ij}=max\left(1-\frac{d_{ij}}{d_{0}},0\right),$$

**Figure 4:**
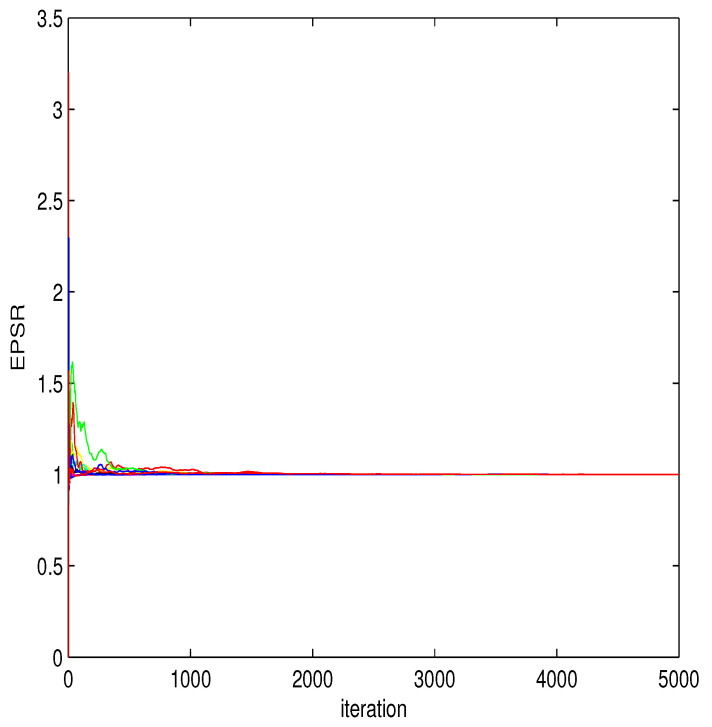
EPSR values for all parameters in Boston data analysis.

**Figure 5:**
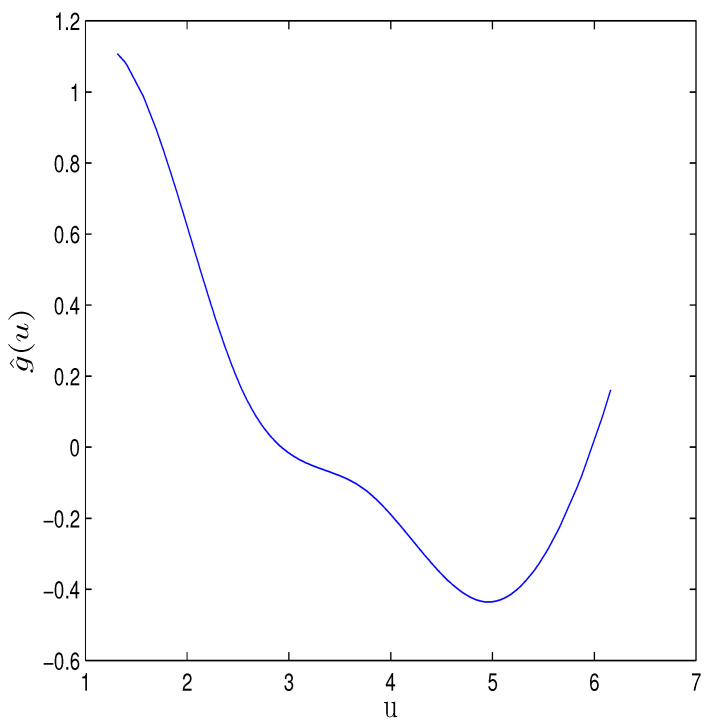
The solid curve is the estimated function of $\hat{g}\left(u\right)$.

**Figure 6:**
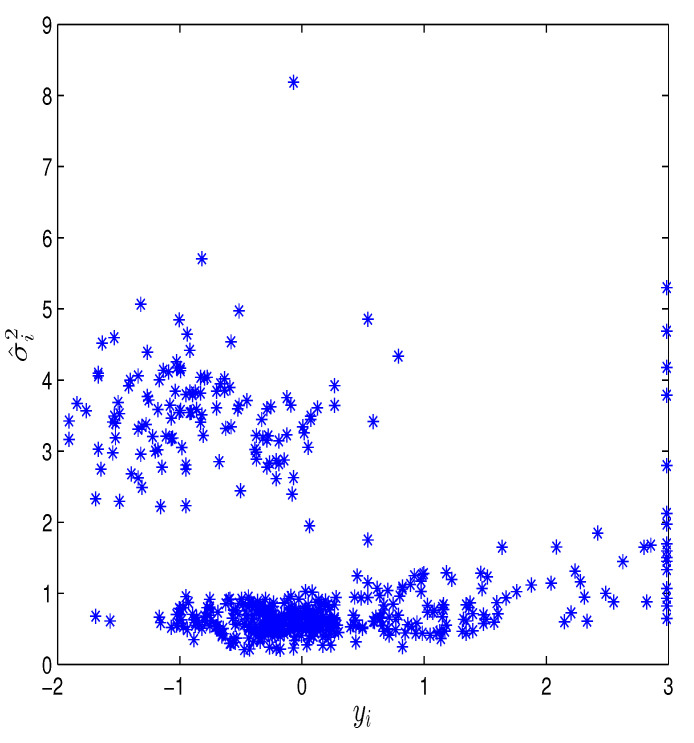
The scatter plot of ${\hat{\sigma }}_{i}^{2}$.

$d_{ij}$ is the Euclidean distance, and $d_{0}$ takes a value of 0.05 as the threshold distance. Thus, the spatial weight matrix contains 19.1% non-zero elements. Then, here we consider the heterogeneous SSAR model as follows:

(12) $$\left\{\begin{array}{c} Y_{i}=\rho \sum_{j=1}^{n}w_{ij}Y_{j}+\sum_{k=1}^{9}X_{ik}\beta_{k}+g\left(u_{i}\right)+\varepsilon_{i},\\ \sigma_{i}^{2}=\exp\left(\sum_{k=1}^{3}Z_{ik}\gamma_{k}\right),\\ i=1,2,\cdots ,506. \end{array}\right.\;$$

where three explanatory variables $Z_{1}=X_{4},Z_{2}=X_{5},Z_{3}=X_{6}$ are selected in the variance model. Thus, the hybrid algorithm proposed earlier is applied to obtain Bayesian estimates of β’s, γ’s, and ρ’s, in which a B-spline with K=3 and noninformative prior information are used. In order to check the convergence of the algorithm, Figure 4 shows the relationship between the EPSR values of all unknown parameters and iterations, indicating that the algorithm converges after approximately 3000 iterations due to the EPSR values of all unknown parameters being less than 1.1 in approximately 3000 iterations. The Bayesian estimates (EST) of β’s, γ’s and ρ’s and their standard deviation estimates (SD), 95% credible intervals (CI) are calculated. Results are given in Table 6. Figure 5 displays the Bayesian estimate of the nonparametric function g(u), which also confirms a significant nonlinear relationship between housing prices and the variable u. Some useful conclusions can be obtained from the results of the table, which are basically consistent with the research results of other authors. For example, the regression parameter corresponding to $X_{1}$ in the mean model is estimated to be negative, indicating that housing prices will decrease as the per capita crime rate in urban areas increases. The estimated coefficient of $X_{4}$ in the mean model is 0.3899, indicating that as the average number of rooms per dwelling increases, housing prices will also increase. ${\hat{\beta }}_{5}$ is negative, indicating that the greater the weighted distances to five Boston employment centres, the lower the housing price will be. The regression parameter corresponding to $X_{8}$ in the mean model is estimated to be negative, indicating that housing prices will decrease as the pupil–teacher ratio by town increases. The regression parameter corresponding to $Z_{2}$ in the variance model is estimated to be negative, indicating that as the weighted distances to five Boston employment centres increases, the fluctuation of housing prices will also decrease. In addition, based on the estimation of γ, we can obtain the estimated value of $\sigma_{i}^{2}$ and present the scatter plot of ${\hat{\sigma }}_{i}^{2}$ in Figure 6, indicating that heteroscedasticity modeling for this dataset is reasonable.

## 6. Conclusions and Discussion

Heteroscedasticity is a common phenomenon in spatial data modeling and analysis. Therefore, this paper proposes heterogeneous SSAR models, where the variance parameter is modeled as a function of the explanatory variable. Like mean regression modeling, the variance component may also depend on various explanatory variables of interest, so estimating the joint models of the mean and variance becomes the basis for avoiding modeling bias and reducing model complexity. Then, based on the nonparametric components approximated by B-splines, we propose a complete Bayesian analysis of a heterogeneous semiparametric spatial autoregressive models. Based on the Gibbs sampler and Metropolis–Hastings algorithm, an effective MCMC sampling algorithm was developed for posterior inference by generating posterior samples from the posterior distributions. Simulation studies and real data analysis based on Boston housing data are used to illustrate the proposed method. The results show that the proposed Bayesian method has high efficiency and fast computational speed.

In addition, there are several interesting questions worth further research in the future. For example, (i) it is interesting to consider variable selection for the parametric component in the context of heterogeneous spatial autoregressive model; (ii) the model proposed in the paper is also a worthwhile issue to study when there are missing response variables.

## Figures and Tables

**Table 1:** Bayesian estimation results of unknown parameters under different sample sizes and prior information when ρ=0.5.

Type	*n*	Para.	Bias	RMS	SD
I	100	$\beta_{1}$	0.0045	0.0911	0.0910
		$\beta_{2}$	0.0084	0.1004	0.1000
		$\beta_{3}$	0.0079	0.0926	0.0922
		$\gamma_{1}$	0.0053	0.1480	0.1480
		$\gamma_{2}$	0.0126	0.1459	0.1454
		$\gamma_{3}$	0.0215	0.1540	0.1524
		ρ	0.0633	0.0975	0.0741
	200	$\beta_{1}$	0.0031	0.0710	0.0709
		$\beta_{2}$	0.0062	0.0674	0.0672
		$\beta_{3}$	0.0014	0.0696	0.0695
		$\gamma_{1}$	0.0252	0.1215	0.1188
		$\gamma_{2}$	0.0093	0.1294	0.1290
		$\gamma_{3}$	0.0075	0.1080	0.1078
		ρ	0.0149	0.0557	0.0536
	300	$\beta_{1}$	0.0073	0.0554	0.0550
		$\beta_{2}$	0.0075	0.0568	0.0563
		$\beta_{3}$	0.0091	0.0597	0.0590
		$\gamma_{1}$	0.0049	0.0841	0.0840
		$\gamma_{2}$	0.0051	0.1074	0.1073
		$\gamma_{3}$	0.0047	0.0885	0.0884
		ρ	0.0090	0.0448	0.0439
II	100	$\beta_{1}$	0.0153	0.0987	0.0975
		$\beta_{2}$	0.0284	0.1122	0.1085
		$\beta_{3}$	0.0282	0.1083	0.1045
		$\gamma_{1}$	0.0223	0.1719	0.1704
		$\gamma_{2}$	0.0656	0.1866	0.1747
		$\gamma_{3}$	0.0540	0.1864	0.1784
		ρ	0.0532	0.0979	0.0822
	200	$\beta_{1}$	0.0057	0.0685	0.0683
		$\beta_{2}$	0.0025	0.0768	0.0768
		$\beta_{3}$	0.0022	0.0727	0.0727
		$\gamma_{1}$	0.0004	0.1174	0.1174
		$\gamma_{2}$	0.0122	0.1401	0.1396
		$\gamma_{3}$	0.0008	0.1202	0.1202
		ρ	0.0320	0.0622	0.0533
	300	$\beta_{1}$	0.0010	0.0658	0.0658
		$\beta_{2}$	0.0036	0.0625	0.0624
		$\beta_{3}$	0.0042	0.0593	0.0591
		$\gamma_{1}$	0.0018	0.1085	0.1085
		$\gamma_{2}$	0.0077	0.1170	0.1167
		$\gamma_{3}$	0.0083	0.0864	0.0860
		ρ	0.0158	0.0439	0.0409
III	100	$\beta_{1}$	0.0001	0.0994	0.0994
		$\beta_{2}$	0.0117	0.1106	0.1099
		$\beta_{3}$	0.0200	0.1073	0.1054
		$\gamma_{1}$	0.0330	0.1788	0.1758
		$\gamma_{2}$	0.0059	0.1855	0.1854
		$\gamma_{3}$	0.0218	0.1882	0.1869
		ρ	0.0553	0.0986	0.0816
	200	$\beta_{1}$	0.0013	0.0686	0.0686
		$\beta_{2}$	0.0100	0.0780	0.0773
		$\beta_{3}$	0.0062	0.0731	0.0729
		$\gamma_{1}$	0.0217	0.1208	0.1189
		$\gamma_{2}$	0.0367	0.1465	0.1418
		$\gamma_{3}$	0.0135	0.1246	0.1238
		ρ	0.0329	0.0624	0.0531
	300	$\beta_{1}$	0.0052	0.0663	0.0661
		$\beta_{2}$	0.0014	0.0627	0.0627
		$\beta_{3}$	0.0014	0.0593	0.0593
		$\gamma_{1}$	0.0121	0.1109	0.1102
		$\gamma_{2}$	0.0233	0.1205	0.1182
		$\gamma_{3}$	0.0160	0.0878	0.0863
		ρ	0.0160	0.0440	0.0410

**Table 2:** Bayesian estimation results of unknown parameters under different sample sizes and prior information when ρ=0.

*Type*	*n*	Para.	Bias	RMS	SD
I	100	$\beta_{1}$	0.0017	0.1025	0.1025
		$\beta_{2}$	0.0065	0.1031	0.1029
		$\beta_{3}$	0.0192	0.0955	0.0936
		$\gamma_{1}$	0.0114	0.1689	0.1685
		$\gamma_{2}$	0.0089	0.1785	0.1783
		$\gamma_{3}$	0.0020	0.1573	0.1573
		ρ	0.0092	0.0934	0.0929
	200	$\beta_{1}$	0.0074	0.0623	0.0618
		$\beta_{2}$	0.0002	0.0833	0.0833
		$\beta_{3}$	0.0042	0.0665	0.0663
		$\gamma_{1}$	0.0001	0.0995	0.0995
		$\gamma_{2}$	0.0050	0.1196	0.1195
		$\gamma_{3}$	0.0177	0.1082	0.1067
		ρ	0.0043	0.0702	0.0701
	300	$\beta_{1}$	0.0038	0.0551	0.0549
		$\beta_{2}$	0.0093	0.0590	0.0582
		$\beta_{3}$	0.0102	0.0442	0.0430
		$\gamma_{1}$	0.0004	0.0904	0.0904
		$\gamma_{2}$	0.0061	0.1070	0.1068
		$\gamma_{3}$	0.0031	0.0977	0.0977
		ρ	0.0004	0.0540	0.0540
II	100	$\beta_{1}$	0.0180	0.1001	0.0985
		$\beta_{2}$	0.0027	0.1205	0.1205
		$\beta_{3}$	0.0111	0.1139	0.1134
		$\gamma_{1}$	0.0456	0.1827	0.1770
		$\gamma_{2}$	0.0596	0.2049	0.1960
		$\gamma_{3}$	0.0397	0.1852	0.1809
		ρ	0.0125	0.0975	0.0967
	200	$\beta_{1}$	0.0000	0.0567	0.0567
		$\beta_{2}$	0.0041	0.0763	0.0762
		$\beta_{3}$	0.0044	0.0598	0.0597
		$\gamma_{1}$	0.0101	0.1219	0.1215
		$\gamma_{2}$	0.0028	0.1599	0.1598
		$\gamma_{3}$	0.0041	0.1352	0.1351
		ρ	0.0012	0.0680	0.0680
	300	$\beta_{1}$	0.0043	0.0525	0.0523
		$\beta_{2}$	0.0004	0.0601	0.0601
		$\beta_{3}$	0.0018	0.0503	0.0503
		$\gamma_{1}$	0.0079	0.0910	0.0907
		$\gamma_{2}$	0.0019	0.1130	0.1130
		$\gamma_{3}$	0.0021	0.1001	0.1000
		ρ	0.0032	0.0663	0.0662
III	100	$\beta_{1}$	0.0016	0.0991	0.0991
		$\beta_{2}$	0.0157	0.1237	0.1227
		$\beta_{3}$	0.0013	0.1135	0.1135
		$\gamma_{1}$	0.0091	0.1838	0.1836
		$\gamma_{2}$	0.0025	0.2074	0.2074
		$\gamma_{3}$	0.0044	0.1888	0.1888
		ρ	0.0117	0.0964	0.0957
	200	$\beta_{1}$	0.0070	0.0573	0.0569
		$\beta_{2}$	0.0033	0.0768	0.0767
		$\beta_{3}$	0.0003	0.0604	0.0604
		$\gamma_{1}$	0.0142	0.1229	0.1220
		$\gamma_{2}$	0.0281	0.1658	0.1634
		$\gamma_{3}$	0.0096	0.1387	0.1384
		ρ	0.0017	0.0679	0.0679
	300	$\beta_{1}$	0.0002	0.0523	0.0523
		$\beta_{2}$	0.0053	0.0605	0.0603
		$\beta_{3}$	0.0044	0.0506	0.0504
		$\gamma_{1}$	0.0067	0.0923	0.0921
		$\gamma_{2}$	0.0179	0.1156	0.1142
		$\gamma_{3}$	0.0071	0.1021	0.1018
		ρ	0.0035	0.0659	0.0658

**Table 3:** Bayesian estimation results of unknown parameters under different sample sizes and prior information when ρ=−0.5.

*Type*	*n*	Para.	Bias	RMS	SD
I	100	$\beta_{1}$	0.0184	0.1006	0.0989
		$\beta_{2}$	0.0087	0.1127	0.1123
		$\beta_{3}$	0.0051	0.1000	0.0999
		$\gamma_{1}$	0.0018	0.1711	0.1710
		$\gamma_{2}$	0.0183	0.1643	0.1633
		$\gamma_{3}$	0.0107	0.1699	0.1695
		ρ	0.0364	0.1197	0.1141
	200	$\beta_{1}$	0.0124	0.0743	0.0733
		$\beta_{2}$	0.0184	0.0786	0.0764
		$\beta_{3}$	0.0022	0.0774	0.0773
		$\gamma_{1}$	0.0182	0.1269	0.1256
		$\gamma_{2}$	0.0091	0.1236	0.1233
		$\gamma_{3}$	0.0024	0.1247	0.1247
		ρ	0.0077	0.0707	0.0703
	300	$\beta_{1}$	0.0078	0.0592	0.0587
		$\beta_{2}$	0.0067	0.0618	0.0614
		$\beta_{3}$	0.0016	0.0549	0.0549
		$\gamma_{1}$	0.0026	0.1008	0.1008
		$\gamma_{2}$	0.0074	0.1121	0.1119
		$\gamma_{3}$	0.0021	0.1044	0.1044
		ρ	0.0099	0.0596	0.0588
II	100	$\beta_{1}$	0.0023	0.1156	0.1156
		$\beta_{2}$	0.0072	0.1083	0.1080
		$\beta_{3}$	0.0073	0.0958	0.0955
		$\gamma_{1}$	0.0519	0.1788	0.1711
		$\gamma_{2}$	0.0447	0.1885	0.1831
		$\gamma_{3}$	0.0173	0.1759	0.1751
		ρ	0.0103	0.1074	0.1069
	200	$\beta_{1}$	0.0013	0.0756	0.0756
		$\beta_{2}$	0.0152	0.0888	0.0875
		$\beta_{3}$	0.0086	0.0740	0.0735
		$\gamma_{1}$	0.0296	0.1097	0.1056
		$\gamma_{2}$	0.0352	0.1359	0.1313
		$\gamma_{3}$	0.0275	0.1190	0.1158
		ρ	0.0039	0.0693	0.0692
	300	$\beta_{1}$	0.0005	0.0520	0.0520
		$\beta_{2}$	0.0082	0.0668	0.0663
		$\beta_{3}$	0.0059	0.0627	0.0625
		$\gamma_{1}$	0.0224	0.1046	0.1022
		$\gamma_{2}$	0.0230	0.1092	0.1068
		$\gamma_{3}$	0.0157	0.0989	0.0977
		ρ	0.0059	0.0662	0.0660
III	100	$\beta_{1}$	0.0143	0.1080	0.1071
		$\beta_{2}$	0.0106	0.1198	0.1194
		$\beta_{3}$	0.0110	0.1107	0.1101
		$\gamma_{1}$	0.0032	0.1955	0.1954
		$\gamma_{2}$	0.0074	0.2199	0.2198
		$\gamma_{3}$	0.0143	0.2213	0.2208
		ρ	0.0321	0.1208	0.1165
	200	$\beta_{1}$	0.0041	0.0691	0.0690
		$\beta_{2}$	0.0002	0.0824	0.0824
		$\beta_{3}$	0.0016	0.0792	0.0791
		$\gamma_{1}$	0.0116	0.1109	0.1103
		$\gamma_{2}$	0.0117	0.1396	0.1391
		$\gamma_{3}$	0.0075	0.1269	0.1267
		ρ	0.0131	0.0798	0.0787
	300	$\beta_{1}$	0.0123	0.0608	0.0595
		$\beta_{2}$	0.0033	0.0655	0.0654
		$\beta_{3}$	0.0008	0.0553	0.0553
		$\gamma_{1}$	0.0055	0.0996	0.0994
		$\gamma_{2}$	0.0042	0.1384	0.1383
		$\gamma_{3}$	0.0247	0.0988	0.0956
		ρ	0.0037	0.0524	0.0522

**Table 4:** The estimate for the nonparametric components based on RASE.

ρ	*n*	Type=I	Type=II	Type=III
0.5	100	0.0456	0.0507	0.0490
	200	0.0302	0.0381	0.0379
	300	0.0230	0.0206	0.0204
0	100	0.0557	0.0616	0.0608
	200	0.0356	0.0379	0.0378
	300	0.0221	0.0251	0.0250
−0.5	100	0.0429	0.0630	0.0534
	200	0.0342	0.0316	0.0355
	300	0.0268	0.0251	0.0214

**Table 5:** Detailed description of relevant variables involved in Boston housing data.

Related Variables	Detailed Description
CRIM	Per capita crime rate by town
ZN	Proportion of residential land zoned for lots over 25,000 sq.ft.
INDUS	Proportion of non-retail business acres per town
CHAS	Charles River dummy variable (= 1 if tract bounds river; 0 otherwise)
NOX	Nitric oxide concentration (parts per 10 million)
RM	Average number of rooms per dwelling
AGE	Proportion of owner-occupied units built prior to 1940
DIS	Weighted distances to five Boston employment centres
RAD	Index of accessibility to radial highways
TAX	Full-value property-tax rate per $10,000
PTRATIO	Pupil–teacher ratio by town
B	$1000{\left(Bk-0.63\right)}^{2}$ where Bk is the proportion of blacks by town
LSTAT	% lower status of the population
MEDV	Median value of owner-occupied homes in USD 1000’s

**Table 6:** Bayesian estimation results in Boston data analysis.

Parameter	EST	SD	CI
$\beta_{1}$	−0.1335	0.1098	(−0.3460, 0.0835)
$\beta_{2}$	0.0386	0.0507	(−0.0614, 0.1397)
$\beta_{3}$	−0.0839	0.0850	(−0.2541, 0.0803)
$\beta_{4}$	0.3899	0.0931	(0.2034, 0.5633)
$\beta_{5}$	−0.1591	0.0693	(−0.3043, −0.0305)
$\beta_{6}$	0.2403	0.1182	(0.0122, 0.4697)
$\beta_{7}$	−0.2143	0.0914	(−0.3908, −0.0346)
$\beta_{8}$	−0.1288	0.0530	(−0.2366, −0.0259)
$\beta_{9}$	0.1058	0.07521	(−0.0437, 0.2515)
$\gamma_{1}$	0.2132	0.1602	(−0.1234, 0.5461)
$\gamma_{2}$	−0.3000	0.1937	(−0.7381, 0.0715)
$\gamma_{3}$	0.6484	0.1974	(0.2865, 1.0527)
ρ	0.1553	0.0910	(−0.0335, 0.3302)

## Data Availability

The data presented in this study are available on request from the corresponding author.
